# Multi Omics Analysis Revealed a Resistance Mechanism of Tibetan Barley (*Hordeum vulgare* L., Qingke) Infected by *Ustilago hordei*

**DOI:** 10.3390/plants12010157

**Published:** 2022-12-29

**Authors:** Juan Li, Jixiang Zhang, Tao Wu, Pei Liu, Pu Li, Xiaobo Yao, Hechun Liu, Yangla Ciren

**Affiliations:** 1 Institute of Agro-Products Processing Science and Technology, Sichuan Academy of Agricultural Sciences, Chengdu 610011, China; 2 State Key Laboratory of Food Nutrition and Safety, Tianjin University of Science and Technology, Tianjin 300457, China; 3 Tibet Academy of Agriculture and Animal Husbandry Sciences, Lhasa 850031, China

**Keywords:** Tibetan barley, *Ustilago hordei*, biological profile, metabolome, proteome, transcriptome

## Abstract

Tibetan barley (*Hordeum vulgare* L., qingke) is the principal cereal cultivated on Tibet. *Ustilago hordei* causing covered smut is a serious disease that limits the yield of qingke. Here, based on multi omics study including metabolome, proteome and transcriptome, we show that during infection, primary metabolisms such as carbohydrate, amino acid, and lipids were significantly changed. Jasmonic acid, which perform as a biotic stress signaler, was significantly repressed, and related genes or proteins also showed different expression in infected qingke. In addition, other defense-related compounds such as riboflavin, ascorbic acid, and protease inhibitors were also detected in omics data. Our results revealed a preliminary biological profile of qingke infected by *U. hordei* and provide a resource for further research.

## 1. Introduction

Barley (*Hordeum vulgare* L.) is one of the important crops in the world that contributes 7% of global production [1]. Tibetan barley (*H. vulgare* L., qingke), a six-rowed hulless barely, is the principal cereal cultivated on the Tibetan Plateau and has been used as a traditional staple food for Tibetans. The disease is an important factor affecting the yield of barley. *Ustilago hordei*, a fungal pathogen causing covered smut, is one of several serious diseases that limit the yield of barley. Infected grain shows black powder, and 100% yield per spike would be lost in qingke. Meanwhile, the annual average loss of 2–5% has been reported due to covered smut [2].

Plants are threatened by different kinds of biotic stresses in natural environment and rely on innate immunity to protect themselves [3]. The physical barrier is the first defense line such as cell wall and the cytoskeleton to deter pathogens [3,4]. The second line is that plant could recognize certain pathogens molecular patterns, which called pathogen-associated molecular patterns (PAMP). Pathogens interact with plant surface by PAMP or translocate effector proteins to the host cell, resulting in signal transduction cascades and activation of resistance genes [5,6]. PAMP-triggered immunity (PTI) and effector-triggered immunity (ETI) composed the plant’s innate immunity system. Cytoskeletal reorganization, cell wall fortification, the generation of reactive oxygen species (ROS), and the synthesis of phytoalexins were induced during the early response, and hypersensitive response (HR) induced in later defense response caused programmed cell death (PCD) to limit pathogen spread [7].

The regulation and execution of both PTI and ETI proceed via the biosynthesis of small metabolic signals such as salicylic acid (SA), jasmonic acid (JA) [8]. High levels of SA and its methyl ester cause the production of pathogen-related (PR) proteins, including chitinases and other hydrolytic enzymes. SA also takes an important role in HR to defense pathogens [9]. JA, which is produced from linolenic acid in chloroplasts and peroxisomes, plays a significant role in inducing plants against necrotrophic and hemibiotrophic pathogens [9]. During pathogen infection, JA and JA-isoleucine can be rapidly induced to active major secondary metabolites and protein expression involved in defense response such as alkaloids, terpenoids, phenylpropane, amino acid derivatives, anti-nutritional proteins, and some pathogen-related proteins [10]. Moreover, the SA- and JA-signaling pathways could negatively influence each other [11]. Some vitamins, such as thiamine (TH), riboflavin (RF), folic acid (FA), and ascorbic acid (AA), also were reported to be linked with defense response [12]. Thiamine is able to activate almost all innate-defense mechanisms, including HR, ROS, callose, PR protein, and phytoalexins, RF could activate the majority defense except HR, while FA induces mainly PR proteins [12].

Energy is critical during plant defense responses due to the expression of genes from multiple defense pathways, and the role of primary metabolism during plant–pathogen interactions is suggested to support cellular energy requirements for plant defense responses [13]. *Arabidopsis* mutant that constitutively expressed defense responses showed stunted development and decreased fertility [14]. In addition to energy, primary metabolites perform as molecules signaling to trigger defense response by signal transduction and pathogen recognition processes such as carbohydrates, proteins, and lipids [15]. For example, Hypersenescence1 (HYS1) mutant has altered sensitivity to sugars or sugar signaling that is mediated by hexokinaseits [16]. Constitutive expresser of PR genes 5 (CPR5), allelic gene of HYS1, mutant shows constitutive pathogen defense responses such as ROS and elevated levels of salicylic acid [17]. Extensive crosstalk between SA, ET, and JA signaling pathways provides the potential for efficient energy allocation [18]. 

In order to have a comprehensive understanding of molecular mechanism and further detail of interaction between *U. hordei* infected and the defense response of qingke that is still unknown, we preform multiple omics analysis for qingke infected by *U. hordei* in this study and could provide a resource for further research.

## 2. Results

### 2.1. Metabolome Profile of Infected Qingke

**Figure 1:**
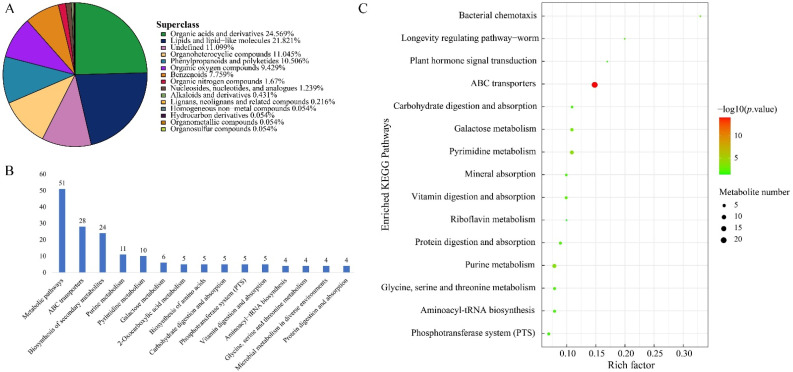
Annotation and enrichment analyses of *U. hordei* responsive metabolome. (**A**) statistic of identified metabolites. Different superclasses show in various colors; (**B**) statistic of annotated KEGG pathways of metabolites; (**C**) KEGG enrichment analysis, hypergeometric tests were performed based on the KEGG classification of significant different metabolites. Abscissa is a rich factor which is defined as the ratio of different metabolites number and the number of genes has been annotated in this pathway; point size represents metabolite number; *p* value, shown by color from green to red.

Untargeted metabolomics was applied to detect metabolic variations in susceptible qingke based on LC-MS. The control and treatment groups each had 12 repetitions. The total ion chromatogram (TIC) and PCA diagram of the samples showed the stability of the device and repeatability of samples (Figure S1). A total of 1856 classes of metabolites were detected. Organic acids and derivatives and lipids and lipid-like molecules were the top two superclasses, accounting for 24.57% and 21.82%, respectively (Figure 1A).

The filter of significantly different metabolites based on OPLS-DA method found a variable importance for the projection (VIP) > 1 and *p* value < 0.05. A total of 187 significant variations in metabolic were detected, including 82 down-regulation and 105 up-regulation. Acetylcarnitine was the maximum down-regulated metabolites, and 4′–methyl-n-methyl hexanophenone was the maximum up-regulated adduct (Table S1). Moreover, lipids and lipid-like molecules including 57 types of adducts (30.5%) were the largest superclass in significant different metabolites (Figure S2).

**Figure 2:**
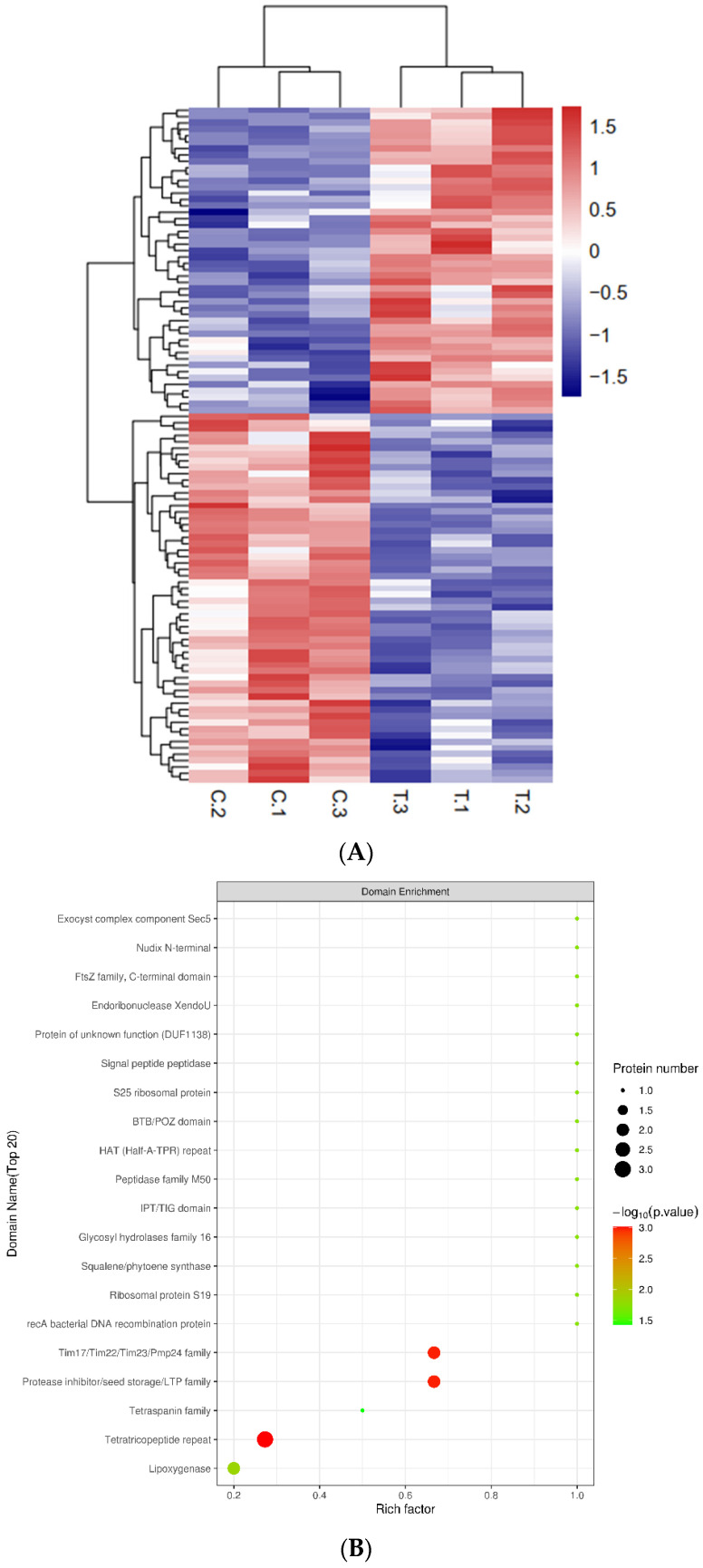

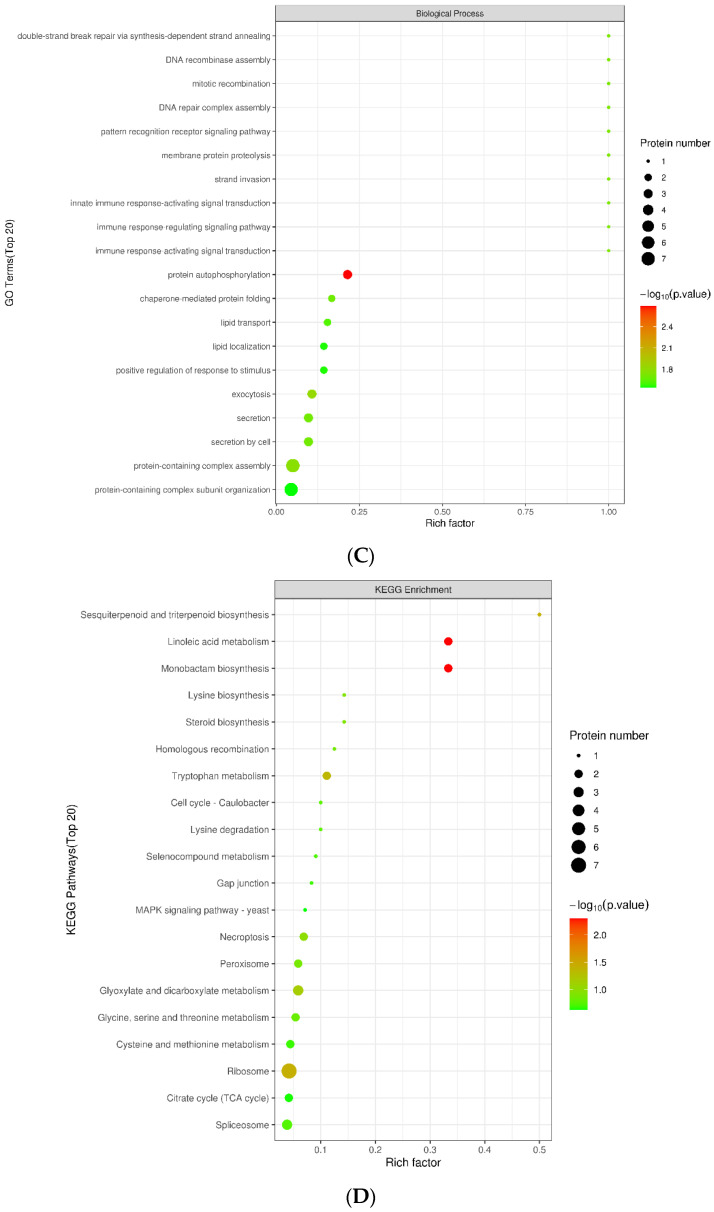
Expression profilling and enrichment of *U. hordei* responsive proteome. (**A**) expression heatmap of differently expressed proteins. The relative expression level of proteins is indicated by color. C: control, T: treat. Hypergeometric tests were performed based on the domain, GO, and KEGG annotation results of DEPs. (**B**) Domain enrichment of DEPs, (**C**) GO enrichment of DEPs in the biological process, (**D**) KEGG enrichment of DEPs. Abscissa is a rich factor which is defined as the ratio of DEP number, and the number of genes has been annotated in this pathway; point size represents DEP number; *p*-value, shown by color from green to red.

KEGG (Kyoto Encyclopedia of Genes and Genomes) was employed to analyze pathways of significant different metabolites. The 187 different metabolites were mapped to 88 KEGG pathways and metabolic pathways had the largest number of adducts (Table S2, Figure 2B). Enrichment analysis showed that 15 pathways were significantly enriched, such as ABC transporters, bacterial chemotaxis, and plant hormone signal transduction (Figure 1C). ABC transporters were the most significant different metabolites, which contain N-acetyl-d-glucosamine and riboflavin that take roles in plant defense. In addition, gibberellin a4 and jasmonic acid, two plant stress response components, were classified into the biosynthesis of secondary metabolites, which was also enriched in the KEGG pathway. Differential abundance score (DA score) was calculated for a total change in ensemble pathways, and six pathways such as glycine, serine, threonine metabolism, and vitamin-related metabolism were more significant than others shown in the results (Figure S3).

### 2.2. Proteome Profile of Infected Qingke

To identify the differently expressed proteins (DEPs) between healthy and infected qingke, we performed a TMT quantitative proteomic analyses. QC results showed that mass deviations of peptides less than 10 ppm, MASCOT score of 72.11% peptides above 20 and abundance ratios between treat and control were close to 1. These indicated the reliability of the pathogen responsive proteome (Figure S4). A total of 5611 proteins were identified, and 5608 of them were quantified, including 58 up-regulated and 48 down-regulated proteins (>1.2 fold change, *p* value < 0.05) that control and treat could be clearly distinguished (Table S3, Figure 2A). Three reported stress-related proteins had been detected based on BlastGO, including ABC1-LIKE KINASE 3 (ABC1K3), RING DOMAIN LIGASE 4 (RGLG4), and AVRPPHB SUSCEPTIBLE 1 (PBS1). ABC1K3 and RGLG4 are reported to be involved in the jasmonic acid pathway [19], while PBS1 is reported toconfers resistance to potyvirus infection in *Arabidopsis* and soybean [20].

To further understand the functions of DEPs, multiple function analysis was applied for proteomic research. Subcellular location analysis showed that DEPs were mostly located in cytoplasm (42) and nucleus (30) (Figure S5). Domain enrichment analysis showed that tetratricopeptide repeat (TPR), protease inhibitor/seed storage/LTP family and Tim17/Tim22/Tim23/Pmp24 family were the top three significant enrichment domains (Figure 2B). Among them, TPR proteins of pathogens have been reported to be related to virulence-associated functions [21]. Protease inhibitor also plays important roles in plant–pathogen interactions [22]. GO classification showed that metabolic process is the main biological process (BP)-related GO term of DEPs, and catalytic activity and cell part/cell were the largest molecular function- and cell component-related GO terms, respectively (Figure S6). GO terms such as protein autophosphorylation, exocytosis and protein-containing complex assembly were significantly enriched in BP (Figure 2C). Meanwhile, cell cortex part and calmodulin-dependent protein kinase activity were the most significant GO terms in CCand MF, respectively (Figure S7).

In addition, KEGG analysis showed all DEPs were mainly distributed in ribosome, glyoxylate and dicarboxylate metabolism and spliceosome (Table S4). Monobactam biosynthesis, linoleic acid metabolism, sesquiterpenoid and triterpenoid biosynthesis, tryptophan metabolism, together with ribosome, were significantly enriched pathways (Figure 2D).

Protein-Protein Interaction (PPI) is a useful tool for understanding molecular system metabolism or signaling pathways. PPI result showed that ELF5A-3, AT5G09500 and ATP1 were nodes which had the most connections with others (Figure S8).

### 2.3. Transcriptome Analysis of Infected Qingke

High-throughput sequencing obtained 70.3 Gb clean data and 531,075,012 clean reads (99.14% of raw reads), average Q30 > 94%. A total of 262,166 transcripts and 153,979 unigenes were assembled. The N50 of transcript and unigenes were 2131 and 1220 bp, respectively. 56.21%, 23.33% and 18.72% of unigenes were annotated successfully in NR, Swiss-Prot and Pfam. A total of 79,565 and 6957 unigenes were annotated in GO and KEGG database. Cellular process and metabolism pathway had the largest number of unigenes in GO and KWGG pathway classification, respectively (Table S5).

**Figure 3:**
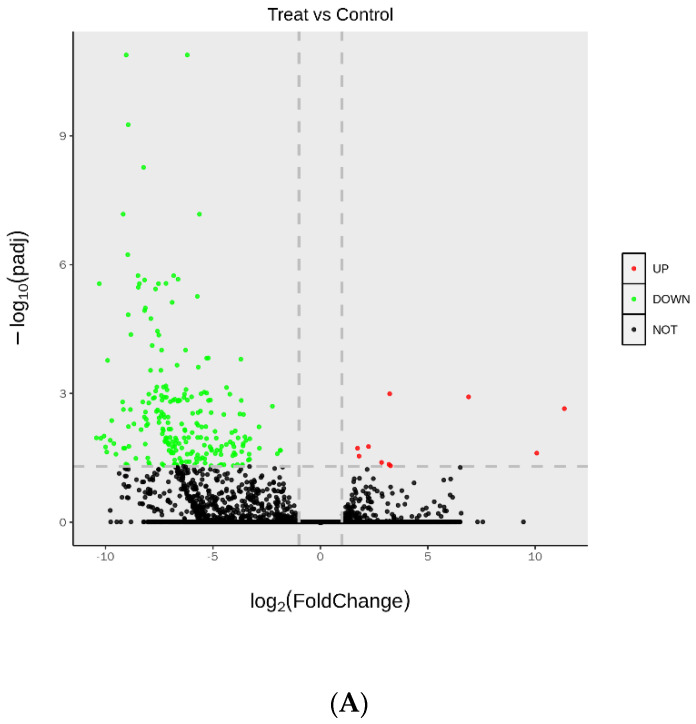

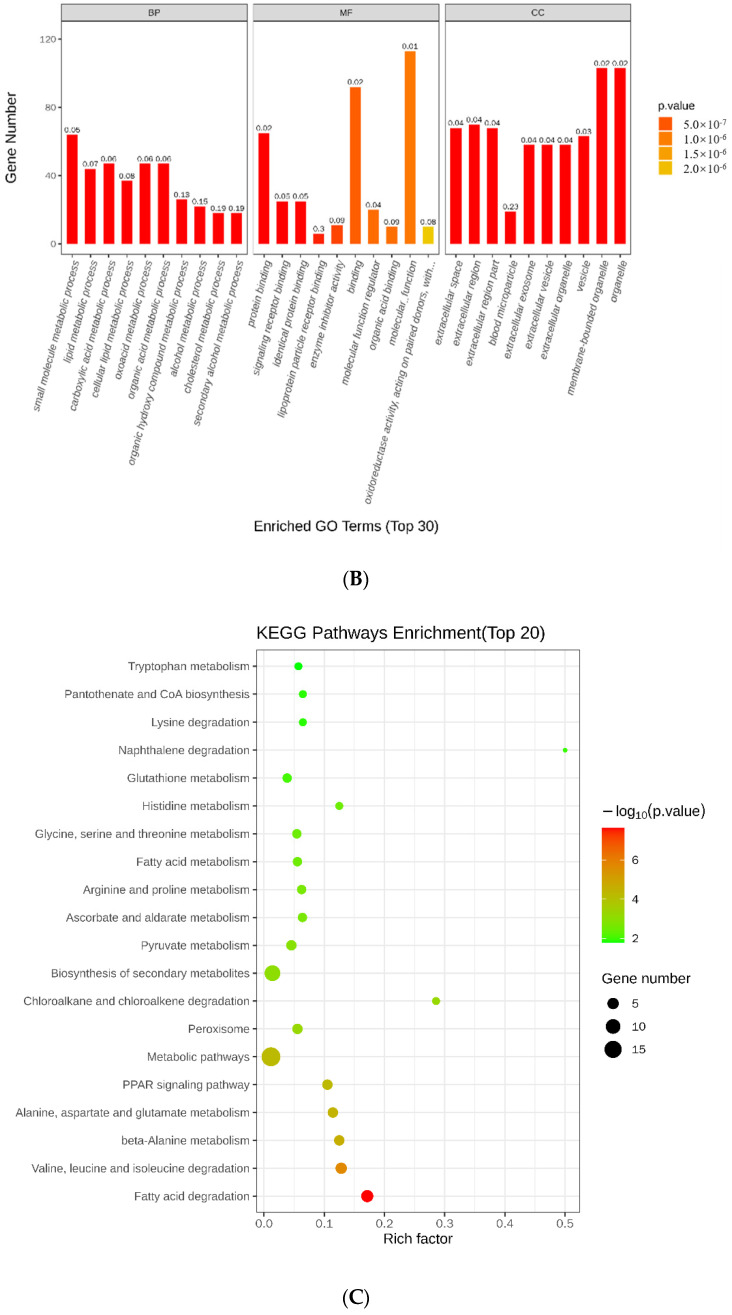
Expression profilling and enrichment of *U. hordei* responsive transcriptome. (**A**) volcano plot of different expression genes. Significantly up–regulated genes are shown by redpoint and down–regulated by green point; (**B**) GO enrichment of DEGs. Hypergeometric tests were performed based on the GO classification of DEGs. Ordinate is gene number, and *p* value of various GO terms are shown by color from orange to red. (**C**) KEGG enrichment of DEGs. Hypergeometric tests were performed based on the KEGG classification of DEGs. Abscissa is rich factor defined as ratio of DEG number and the number of genes has been annotated in this pathway; point size represents DEG number; *p* value, shown by color form green to red.

After 82.86% of clean reads were mapped to the reference sequence assembled by Trinity, fragments Per Kilobase of exon model per Million mapped fragments (FPKM) were calculated to quantify the expression of genes. Overall, a total of 240 different expression genes (DEGs) were detected, including 230 down-regulated and 10 up-regulated genes in infected qingke (Figure 3A, Table S6). GO enrichment showed that DEGs enriched in various metabolic processes, including small molecule, lipid, and oxoacid metabolic process in BP. And results of enrichment in MF indicated binding function was the most common GO term of DEGs (Figure 3B, Table S7). In the KEGG enrichment results, fatty acid degradation was the most significantly enriched pathway, whereas amino acid metabolism-related pathways were the largest enriched term in results indicating primary metabolic pathway, especially amino acid metabolism, was critical for resistance (Figure 3C, Table S8). Moreover, two bZIP transcript factors which reported to be involved in biotic stress were detected in DEGs.

### 2.4. Co-Analysis of Multi Omics

We obtained metabolome, proteome, and transcriptome data between healthy and infected qingke and checked whether they had overlap within GO and KEGG pathways. Results showed that there were 13 overlapped in GO enrichment of proteome and transcriptome, including multiple protein related and lipid related GO terms, secretion and exocytosis in BP, protein binding in MF, cell periphery in CC (Table 1). For KEGG pathways, most pathways of amino acid metabolism and carbohydrate metabolism were overlapped; Cysteine and methionine metabolism, glycine, serine and threonine metabolism, valine, leucine and isoleucine degradation and tryptophan metabolism. Glutathione metabolism in amino acid metabolism, alpha-linolenic acid metabolism in lipid metabolism, drug metabolism—other enzymes in Xenobiotics biodegradation and metabolism were all detected in metabolome, proteome and transcriptome (Table 2). Plant hormone signal transduction, cytochrome P450 and others that related with assistance also co-occurred in two omics at least. These overlapped GOs and pathways may take important roles in defense response of qingke during the infection.

## 3. Discussion

In the process of interaction with pathogens, plants activate their own immunity by responding to the invasion of pathogens and adopt a variety of defense mechanisms to resist the invasion of pests and diseases. In this study, we used multiple omics technology to detected biological changes between health and *U. hordei* infected qingke.

In metabolome, 1856 classes of metabolites were detected. It was found that 187 were significantly different metabolites and organic acids and derivatives, and lipid and lipid-like molecules were the top two function classes of these metabolites. Lipids have significant influences on pathogenesis and could be used as a resistance mechanism during plant–microbe interactions [23]. Literature reported that plant lipid metabolism is the target of pathogens secreted toxins [24]. Plants could adapt defense mechanisms against pathogens using different compounds such as lipopolysaccharides, sphingolipids, and lipid-binding proteins [25]. Regulatory lipids are lipids that mediate of signaling and regulatory cascades and have an effective function at low concentrations [26]. These classes of lipids include polyunsaturated fatty acid derivatives like oxylipins, eicosanoids and jasmonic acid [27,28]. In this study, it was found that jasmonic acid (JA) was significantly up-regulated by the infection of pathogen. JA could crosstalk with other phytohormone including abscisic acid (ABA), ethylene (ET) and salicylic acid (SA) to regulate plant defense against pathogen [29,30,31,32]. For example, JA-mediated defense responses are raised against fungal pathogens like *Botrytis cinerea*. They have been demonstrated to play a role in the defense against some hemibiotrophic pathogens, such as *Xanthomonas oryzae* [33]. Another detected hormone in this study was Gibberellin (GA), which is essential for multiple developmental processes in plants. JA and GA signaling pathways could regulate plant growth and defense response antagonistically that JA defense over growth by interfering with gibberellin signaling cascade [34]. Riboflavin and ascorbic acid, two kinds of vitamin, were also detected in significantly different metabolites identified between heathy and infected qingke. Riboflavin has been reported to protect or induce resistance against various pathogens in different plants such as *Arabidopsis*, tobacco, rice, and soybean [12]. Riboflavin treatment could up-regulate multiple host–defense responses in several plants. A rapid H_2_O_2_ accumulation as a critical step in riboflavin signal transduction during *P. syringae* pv. Tomato DC3000 infected *Arabidopsis* [35]. Moreover, riboflavin could induce expression of genes such as *NPR1* that control systemic acquired resistance [36]. In addition, riboflavin is implicated callose deposition which callose act as physical barrier against pathogen [37,38]. Ascorbic acid is the most abundant cellular antioxidant considered as a major antioxidant compound among the plant antioxidant-defense system [39]. The de novo biosynthesis of ascorbic acid could be stimulated by treatment with methy-ljasmonate in suspension cells of *N. tabacum* and *A. thaliana* [40]. In addition, ascorbic acid is a main precursor of oxalic acid (OA), which also was detected as significantly different metabolites in this study; OA was reported to be involves in the synthesis of H_2_O_2_ and take a critical role in plant-pathogen interaction [41]. N-acetyl-d-glucosamine, which is a basic constituent unit of chitin, showed significant increase in infected qingke. Chitin is an important component of fungal pathogenicity that could be recognized by plants and triggers various defense responses [42]. Moreover, chitin could induce ion efflux and ROS, increased levels of phytoalexins and hypersensitivity in infected cells [43,44]. These results indicated that qingke could elicit by chitin and against *U. hordei* through phytohormones such as JA and vitamins such as riboflavin and ascorbic acid.

For proteome, 106 significantly different proteins were identified. *ABC1K3* and *RGLG4*, homologous genes of TR36261_c0_g2_ORF and TR2862_c2_g1_ORF, were reported to be involved in JA pathway. abc1k3 showed rapid chlorosis upon high light stress, and irreversible, senescence-like phenotype during drought stress and nitrogen limitation, plastid jasmonate biosynthesis enzymes were recruited to the abc1k3 plastoglobules but not wild-type [19]. RGLG4 has ubiquitin ligase activities and expression changes of *RGLG4*, and its homologous *RGLG4* could affect JA-inductive gene expression. Both of them responded to methyl JA, *P. syringae* pv. *tomato* DC3000 and wounding in a COI1-dependent manner [45]. *PBS1* is defense related gene that is a homologous gene of a significantly different protein, TR584_c2_g1_ORF. PBS1 could form a preactivation complex with RESISTANCE TO PSEUDOMONAS SYRINGAE 5 (RPS5) and triggers RPS5 activation upon AvrPphB-dependent cleavage [20]. In domain enrichment analysis, TPR and protease inhibitors were significantly enriched. TPR-containing proteins have been reported to play virulence-associated functions, such as the translocation of virulence factors into host cells, and the blocking of phagolysosomal maturation in bacterial pathogens [21]. *Arabidopsis suppressor* of *rps4-RLD 1* (*SRFR1*), which encodes a conserved tetratricopeptide repeat protein, functions as a negative regulator and could enhance the resistance of bacterial effector AvrRps4 in *srfr1* mutant [45]. Plant protease inhibitors (PI) were also found to take roles in plant immunity through regulation of endogenous plant proteases and inhibition of pathogen proteases [46,47]. PI from barley could act against proteases from *Fusarium culmorum* and the *A. thaliana* unusual serine protease inhibitor could defend against necrotrophic fungi *Botrytis cinerea* and *Alternaria brassicicola* [48]. On the other hand, pathogens may inhibit plant PI from reducing deleterious effects, such as *U. maydis*, which is a closely related species of *U. hordei*, could induce maize cystatin CC9 when infected to inhibit cysteine proteases [49]. These results favorably proved JA was important component in qingke resistance. It suggested TPR family genes and plant protease inhibitors may contribute for *U. hordei* defense. *ELF5A-3*, AT5G09500 and *ATP1*, the homologous of TR5765_c0_g1_ORF, TR4063_c0_g1_ORF_1 and TR41168_c0_g1_ORF, were the top 3 genes within PPI analysis. *EIF5A-3*, homologous of *ELF5A-3*, could regulate programmed cell death caused by the infection of *Pseudomonas syringae* pv. tomato DC3000 (Pst DC3000) [50]. AT5G09500 is a Ribosomal protein S19 family protein that showed expression change in response to *Agrobacterium tumefaciens*, suggested TR5765_c0_g1_ORF and TR4063_c0_g1_ORF_1 may play important roles in qingke.

We also processed transcriptome analyses. The 240 DEGs and GO, KEGG enrichment results were shown in Figure 3. For a more profound and more comprehensive understanding biological change within *U. hordei* infected qingke, we have counted the overlapping parts of GO and KEGG terms in the transcriptome, proteome, and metabolome (Table 1 and Table 2). In the overlapping part of KEGG, primary metabolism was the most abundant pathway, including amino acid metabolism, carbohydrate metabolism and lipid metabolism. Expression of genes involved in carbohydrate metabolism processes such as glycolysis, pentose phosphate pathway and TCA could be induced by pathogens or pathogen-derived elicitors, resulting in downstream defense responses, such as the generation of ROS and the activation of PR genes [7]. For example, *AtHXK1*, which phosphorylates glucose to glucose-6-phosphate, is a positive regulator of *PR-1* and *PR-5* [51]. Moreover, *Nicotiana benthamiana* downregulation of HXK1 expression caused by virus-induced gene silencing could increase the accumulation of H_2_O_2_ and expression of transcripts associated with defence responses result in PCD [52]. Overexpression of the Pyruvate decarboxylase (*PDC*) gene could increase callose deposition and expression of PR genes and impair pathogen spread upon inoculation with *P. infestans* [53]. Amino acid metabolism is another primary metabolism that has much overlapping terms. Some omics papers also reveal amino acid metabolism changes in result, such as valine, leucine, and tyrosine [54]. For example, lht1 (lysine histidine transporter 1) of *Arabidopsis* has reduced contents of glutamine, alanine, and proline compared with wild-type, shows enhanced resistance to diverse bacterial, fungal and oomycete pathogens and exhibited increased callose deposition, higher accumulation of salicylic acid (SA) and constitutive expression of PR-1 [55]. Levels of aspartic acid, threonine and valine showed a decrease and the level of tryptophan showed an increase in our data. Oleic acid, which is classed in fatty acid biosynthesis of lipid metabolism, was detected in metabolome and one gene involved in β-oxidation was detected in the transcriptome. Suppressor of SA-insensitivity (SSI2/FAB2), which catalyzes the desaturation of the stearic acid to oleic acid, its *Arabidopsis ssi2* mutant showed higher expression of the resistance and exhibited spontaneous lesion formation associated with high levels of SA [56]. In addition, linolenic acid was detected in alpha-linolenic acid metabolism of the metabolome. LA was shown to activate the O^2−^-generating enzyme NADPH oxidase. O^2−^ is required for HR in *fad7*/*fad8* double mutant which was unable to suppress the *P. syringae* pv. tomato DC3000 because of insufficient LA [57]. Both proteins and genes involved in LA participated in the JA biosynthesis pathway.

LA is one precursor of JA [34]. TR64995_c0_g3 homologous, *ACYL-COA OXIDASE 1* (*ACX1*), encoded a medium to long-chain acyl-CoA oxidase and was involved in jasmonate biosynthesis; its expression could be induced by abscisic acid, jasmonate and abiotic stress [58,59]. Lipoxygenase 2.1 is blast result of TR3086_c0_g2_ORF, which exhibits linoleate 13-lipoxygenase and arachidonate 15-lipoxygenase activity in barely, could be induced by exogenous application of jasmonic acid methyl [60]. In addition, cytochrome P450 related pathways had overlapped in proteome and transcriptome. Cytochrome P450s (CYPs) are an oxidoreductases class of enzyme and catalyze NADPH/O^2^-dependent hydroxylation [61]. CYPs could protect plants from harsh environmental conditions, including abiotic and biotic stress, by enhancing antioxidant activity [62]. These suggested that primary metabolism, including carbohydrate, amino acid and lipid, were largely changed in infected qingke and these metabolisms may take important roles against *U. hordei*.

Overall, the metabolomic results indicated that qingke might elicit resistance to *U. hordei* through chitin and some phytohormones, such as JA, riboflavin, and ascorbic acid. Proteomic data favorably proved JA was an important component in qingke resistance and suggested TPR family genes and plant protease inhibitors may contribute for *U. hordei* defense. Expressions of TR5765_c0_g1_ORF, TR4063_c0_g1_ORF_1, and TR41168_c0_g1_ORF were changed and may take important roles against *U. hordei*. Transcriptome analysis showed that primary metabolism including carbohydrate, amino acid, and lipid were largely changed in infected qingke, and these metabolisms may take important roles in *U. hordei* defense.

## 4. Materials and Methods

### 4.1. Sample Collection

Diseased and healthy tibetan barley (*Hordeum vulgare* L., qingke) seeds were cultured in the experimental field of Agriculture Research Institute, Tibet Academy of Agriculture and Animal Husbandry Sciences, China. Leaves of health qingke and *U. hordei* infected qingke were sampled as control and treated group during five-leaf stage, respectively. All samples were frozen immediately in liquid nitrogen and stored at −80 °C. Metabolome analysis had 12 biological repeats, proteome and transcriptome had three biological repeats, respectively.

### 4.2. RNA Extraction and Sequencing

Total RNA was extracted using TRIzol^®^ reagents (Invitrogen, Carlsbad, CA, USA) and qualified by 1% agarose gel electrophoresis. Then the RNA quaity and purity were assessed using Nanodrop (ThermoFisher, Waltham, MA, USA) and Bioanalyzer (Agilent 2100, Santa Clara, CA, USA). The RNA samples met all the quality standards (A260/A280 = 1.8–2.2, and RNA integrity number (RIN) > 6.5) were used for cDNA library construction. Libraries were synthesized using TruSeq RNA Library Preparation Kit (Illumina, San Diego, CA, USA) and paired-end (PE) sequenced by Hiseq 4000 (Illumina, San Diego, CA, USA).

### 4.3. Protein Extraction and Digestion

SDT buffer (4% SDS, 100 mM Tris-HCl, 1 mM DTT, H7.6) was used for sample lysis and protein extraction. The BCA Protein Assay Kit (Bio-Rad, Hercules, CA, USA) was used for the quantification of protein content. Protein was digested based on filter-aided sample preparation (FASP) and desalted on C18 Cartridges (Empore™ SPE Cartridges C18 (standard density), then concentrated by vacuum centrifugation and reconstituted in 40 µL of 0.1% (*v*/*v*) formic acid. A total of 100 μg peptide mixture of each sample was labeled using iTRAQ reagent according to the manufacturer’s instructions (Applied Biosystems, Waltham, MA, USA).

### 4.4. LC-MS/MS Analysis

For metabolomics, UHPLC (1290 Infinity LC, Agilent Technologies, Santa Clara, CA, USA) coupled to a quadrupole time-of-flight (AB Sciex TripleTOF 6600) were used. 2.1 mm × 100 mm ACQUIY UPLC BEH 1.7 µm column (waters, Ireland) was used for sample separation. After separation, both electrospray ionization (ESI) positive and negative modes were applied. In order to avoid the influence caused by the fluctuation of the instrument detection signal, a random order was used for continuous analysis of the sample. For ESI source conditions were set as Ion Source Gas1 = 60, Ion Source Gas2 = 60, curtain gas = 30, source temperature: 600 °C, IonSpray Voltage Floating (ISVF) ± 5500 V.

Proteomics was analyzed on a Q Exactive mass spectrometer (Thermo Scientific) that was coupled to Easy nLC (Proxeon Biosystems, now Thermo Fisher Scientific) for 60 min. Reverse phase trap column (Thermo Scientific Acclaim PepMap100, 100 μm × 2 cm, nanoViper C18) that contains peptides connected to the C18-reversed phase analytical column (Thermo Scientific Easy Column, 10 cm long, 75 μm inner diameter, 3 μm resin) in buffer A (0.1% Formic acid) and separated with buffer B (84% acetonitrile and 0.1% formic acid) at a flow rate of 300 nL/min. MS set to positive mode and using data-dependent top10 method to choose the most abundant precursor ions from the survey scan (300–1800 *m*/*z*).

### 4.5. Metabolome Data Processing

The raw MS data (wiff.scan files) were converted to MzXML files by ProteoWizard MSConvert and imported into XCMS software (Scripps Research, La Jolla, CA, USA). CAMERA (Collection of Algorithms of MEtabolite pRofile Annotation) was used for the annotation of isotopes and adducts. Variables having more than 50% of the nonzero measurement values in at least one group were submitted for the following analyses. Compound identification was performed by comparing accuracy *m*/*z* value (<25 ppm). Orthogonal partial least-squares discriminant analysis (OPLS-DA) [63] were performed by R package (ropls). The variable importance in the projection (VIP) value of each variable in the OPLS-DA model was calculated, VIP > 1 and *p* value < 0.05 were used to screen significant changed metabolites.

### 4.6. Proteome Data Processing

The MS raw data were searched using the MASCOT engine (Matrix Science, London, UK; version 2.2) embedded into Proteome Discoverer 1.4 software (Thermo Fisher Scientific, Waltham, MA, USA) for identification and quantitation analyses. The protein ratios are calculated as the median of only unique peptides of the protein. Protein with Fold Change (FC) > 1.2 or <0.83 and *p* value < 0.05 were considered as differentially expressed proteins.

### 4.7. Transcriptome Data Processing

The clean data were assembled by Trinity after quality control. Unigenes were annotated in NR, Swissprot, PFAM, GO, and KO by NCBI blast 2.6.0, Blast2GO v2.5 and KAAS. Clean reads were mapped to transcripts that assembled by Trinity as a reference and expected number of fragments per kilobase of transcript sequence per millions base pairs sequenced (FPKM) calculated by featureCounts were used for analysis. DEseq2 [64] was used for identification of different expression genes (log2Fold Change (FC) > |1|, q value < 0.05).

### 4.8. Cluster Analysis

Cluster 3.0 http://bonsai.hgc.jp/~mdehoon/software/cluster/software.htm (accessed on 12 November 2021) and Java Treeview 1.1.1 (Oracle, Redwood Shore, CA, USA) [65] were employed for hierarchical clustering analysis based on Euclidean distance algorithm.

### 4.9. Subcellular Localization and Function Annotation

CELLO [66] was used to predict protein subcellular localization. Protein domain was identified by InterProScan software. For differentially expressed proteins, NCBI BLAST+ client software (ncbi-blast-2.2.28+-win32.exe) and InterProScan were used to find homologue sequences, then gene ontology (GO) terms were annotated by Blast2GO which also used for annotation of DEGs in transcriptome. The studied proteins and transcripts were blasted against the online Kyoto Encyclopedia of Genes and Genomes (KEGG) database http://geneontology.org/ (accessed on 23 November 2021) to retrieve KEGG terms. Enrichment analysis was applied based on Fisher’s exact test, and Benjamini–Hochberg correction for multiple testing was further applied to adjust derived *p*-values. Moreover, only functional categories and pathways with *p*-values under a threshold of 0.05 were considered significant in metabolomic and proteomic, but q-value in the transcriptome. The protein–protein interaction (PPI) information was searched from IntAct molecular interaction database http://www.ebi.ac.uk/intact/ (accessed on 6 December 2021) and visualized by Cytoscape [67].

## 5. Conclusions

**Figure 4:**
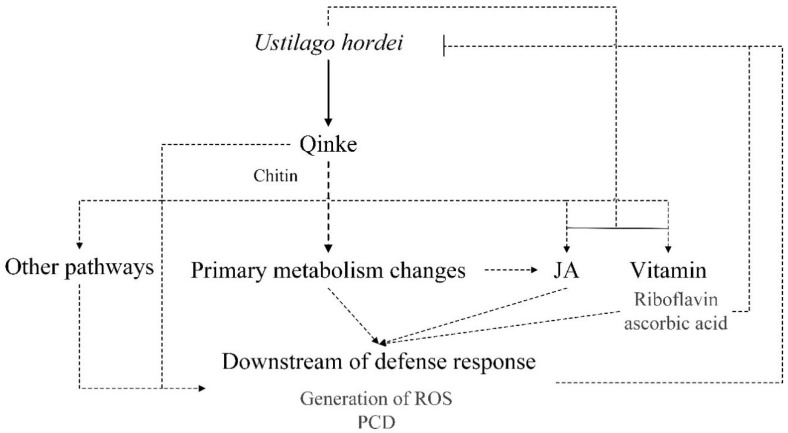
Hypothesis model of qingke interacted with *Ustilago hordei.* Arrow indicated activation and horizontal line indicated repression, dotted line indicated need to further biological evidence in qingke.

In summary, we used multiple omics methods to analyze biological changes in transcript, protein and metabolism levels between healthy gingke and *U. hordei* infected qingke. A model of defense responses of qingke to *U. hordei* was shown in Figure 4. During *U. hordei* infection, qingke may recognize chitin to trigger cascades of activities, including the changes in primary metabolism and JA-related and vitamin-related pathways, then induce downstream defense responses such as ROS and PCD against *U. hordei*, whereas *U. hordei* could repress response compounds, such as JA and ascorbic acid that were downregulated in our data, to ensure spread. Our study revealed a comprehensive omics profile of qingke infected by *U. hordei* and provided a resource for further research of covered smut.

## Figures and Tables

**Table 1:** Statistics of overlapping GO term in proteomic and transcriptome.

GO ID	Term	Category
GO: 0022607	cellular component assembly	BP
GO: 0006887	exocytosis	BP
GO: 0010876	lipid localization	BP
GO: 0006869	lipid transport	BP
GO: 0048584	positive regulation of response to stimulus	BP
GO: 0006468	protein phosphorylation	BP
GO: 0065003	protein-containing complex assembly	BP
GO: 0043933	protein-containing complex subunit organization	BP
GO: 0080134	regulation of response to stress	BP
GO: 1901700	response to oxygen-containing compound	BP
GO: 0046903	secretion	BP
GO: 0032940	secretion by cell	BP
GO: 0071944	cell periphery	CC
GO: 0005515	protein binding	MF

The BP, CC, and MF are the abbreviations of biological process, cellular component, and molecular function, respectively.

**Table 2:** Statistics of overlapping KEGG pathways.

Pathway	KEGG ID	Metabolomic	Proteomic	Transcriptome
**Amino acid metabolism**				
Cysteine and methionine metabolism	ko00270	Y	Y	Y
Glycine, serine and threonine metabolism	ko00260	Y	Y	Y
Tryptophan metabolism	ko00380	Y	Y	Y
Valine, leucine and isoleucine degradation	ko00280	Y	Y	Y
Lysine degradation	ko00310		Y	Y
Alanine, aspartate and glutamate metabolism	ko00250	Y		Y
Arginine biosynthesis	ko00220	Y		Y
Histidine metabolism	ko00340	Y		Y
Tyrosine metabolism	ko00350	Y		Y
Lysine biosynthesis	ko00300	Y	Y	
**Biosynthesis of other secondary metabolites**				
Monobactam biosynthesis	ko00261	Y	Y	
Phenylpropanoid biosynthesis	ko00940	Y		Y
**Carbohydrate metabolism**				
Amino sugar and nucleotide sugar metabolism	ko00520	Y		Y
Ascorbate and aldarate metabolism	ko00053	Y		Y
Galactose metabolism	ko00052	Y		Y
Glycolysis/Gluconeogenesis	ko00010		Y	Y
Glyoxylate and dicarboxylate metabolism	ko00630		Y	Y
Pentose and glucuronate interconversions	ko00040	Y		Y
Propanoate metabolism	ko00640		Y	Y
Pyruvate metabolism	ko00620		Y	Y
Starch and sucrose metabolism	ko00500	Y		Y
**Cell growth and death**				
Ferroptosis	ko04216		Y	Y
**Energy metabolism**				
Carbon fixation in photosynthetic organisms	ko00710	Y	Y	
Methane metabolism	ko00680		Y	Y
**Environmental adaptation**				
Thermogenesis	ko04714	Y		Y
**Global and overview maps**				
Biosynthesis of amino acids	ko01230	Y		Y
Biosynthesis of secondary metabolites	ko01110	Y		Y
Carbon metabolism	ko01200	Y		Y
Degradation of aromatic compounds	ko01220	Y		Y
Metabolic pathways	ko01100	Y		Y
Microbial metabolism in diverse environments		Y		Y
**Lipid metabolism**				
alpha-Linolenic acid metabolism	ko01120	Y	Y	Y
Biosynthesis of unsaturated fatty acids	ko01040	Y		Y
Fatty acid biosynthesis	ko00592	Y		Y
Steroid biosynthesis	ko01040	Y	Y	
**Metabolism of cofactors and vitamins**				
Pantothenate and CoA biosynthesis	ko00770	Y		Y
**Metabolism of other amino acids**				
beta-Alanine metabolism	ko00410	Y		Y
Glutathione metabolism	ko00480	Y	Y	Y
**Metabolism of terpenoids and polyketides**				
Limonene and pinene degradation	ko00903	Y		Y
**Nucleotide metabolism**				
Purine metabolism	ko00230	Y	Y	
Pyrimidine metabolism	ko00240	Y		Y
**Signal transduction**				
cAMP signaling pathway	ko04024	Y		Y
cGMP-PKG signaling pathway	ko04022	Y	Y	
HIF-1 signaling pathway	ko04066	Y	Y	
Plant hormone signal transduction	ko04075	Y		Y
**Transport and catabolism**				
Peroxisome	ko04146		Y	Y
**Xenobiotics biodegradation and metabolism**				
Drug metabolism—cytochrome P450	ko00982		Y	Y
Drug metabolism—other enzymes	ko00983	Y	Y	Y
Metabolism of xenobiotics by cytochrome P450	ko00980		Y	Y

The Y is the abbreviation of “Yes”, means the pathways was annotated.

## Data Availability

Not applicable.

## Supplementary Materials

The following supporting information can be downloaded at: https://www.mdpi.com/article/10.3390/plants12010157/s1, Figure S1. (A) and (B) are total ion chromatograms of positive ion model and negative ion model, respectively; (C) and (D) are PCA analysis plots of positive ion model and negative ion model, respectively; Figure S2: Fold change plot of significantly different metabolites. (A) positive ion model; (B) negative ion model; up-regulation and down-regulation are shown by red and green. The different colored words represent different superclasses of metabolites; Figure S3: Differential abundance score. The x-axis is DA score, which represents total changes for all metabolites in a metabolic pathway; plus means up-regulation and minus means down-regulation. The length of the line represents the absolute Da score, and the dot size of the end point of the line represents the number of metabolites in that pathway. Shades of color of point are proportional to DA score; Figure S4: QC of proteome. (A) Mass error distribution. (B) Ion score distribution. (C) Ratio distribution between treat and control; Figure S5: Subcellular localization statistic of DEPs. Number represents number of DEPs; Figure S6: GO statistic of DEPs. Biological process, molecular function and cellular component were distinguished by different colors; Figure S7: GO enrichment analysis of proteome. (A) Molecular function; (B) cell component. Abscissa is rich factor defined as ratio of DEP number, and the number of genes was annotated in this pathway; point size represents DEP number; *p* value shown by color from green to red; Figure S8: Protein–protein interaction network. Circle nodes indicate differentially expressed proteins, and lines indicate protein–protein interactions. Red indicates up-regulated protein, and blue is down-regulated protein. Circle size indicates the degree of protein connectivity. Table S1: List of metabonomics significant differences; Table S2: Annotation and enrichment list of metabolite kegg; Table S3: List of significant differences in proteomics; Table S4: Annotation and enrichment list of protein kegg; Table S5: Qualitative and quantitative list of transcriptomics; Table S6: List of significant difference gene annotations; Table S7: GO annotation list of significant difference genes; Table S8: KEGG annotation list of significant difference genes.
