# Supplementary material for: Multi Omics Analysis Revealed a Resistance Mechanism of Tibetan Barley (Hordeum vulgare L., Qingke) Infected by Ustilago hordei
Source: Plants (Basel). 2022 Dec 29;12(1):157. doi: 10.3390/plants12010157 (PMC9824760; doi:10.3390/plants12010157)
Supplement: Supplementary file 1 [file plants-12-00157-s001.zip › plants-1905294-supplementary figures.pdf]

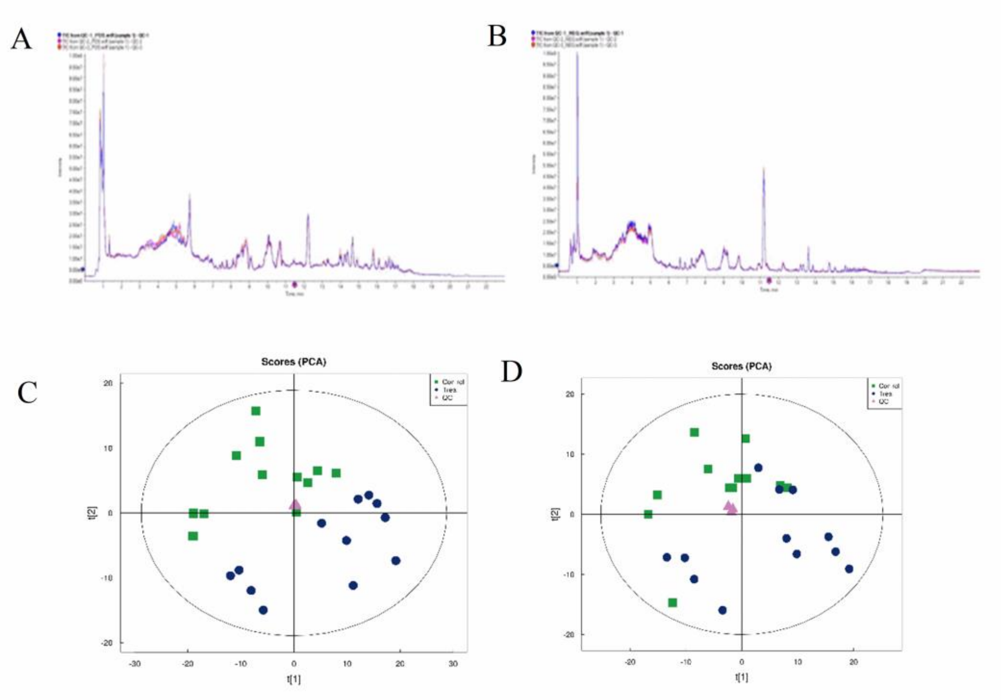

Figure S1. (**A**) and (**B**) are total ion chromatograms of positive ion model and negative ion model, respectively; (**C**) and (**D**) are PCA analysis plots of positive ion model and negative ion model, respectively.

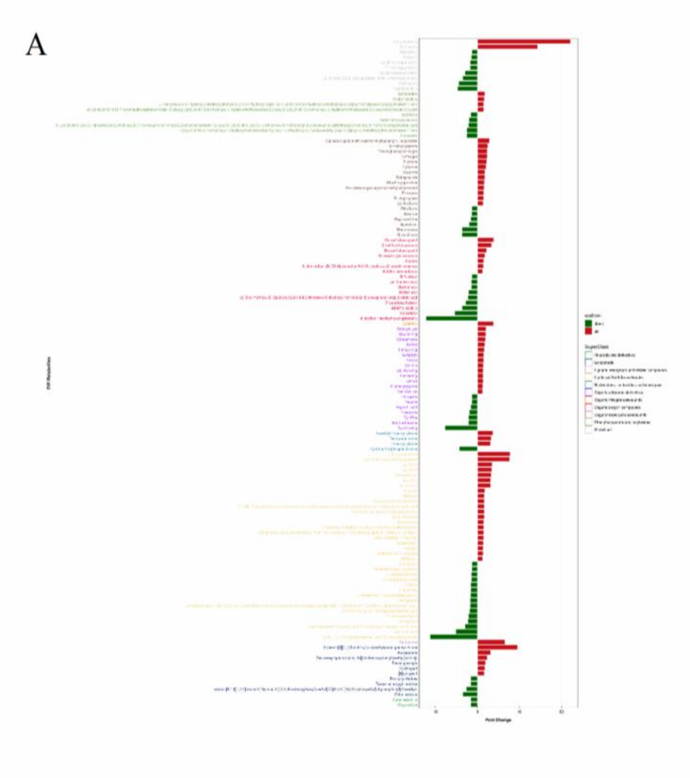

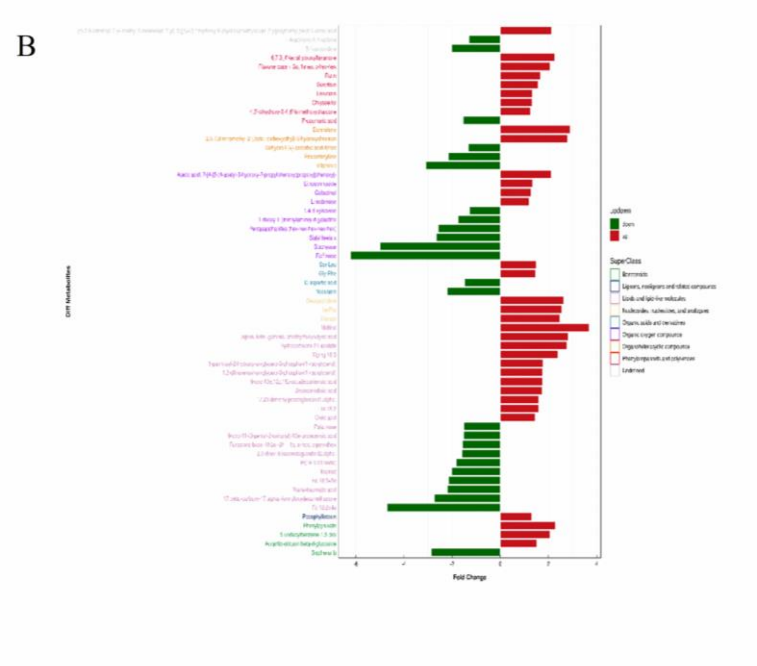

Figure S2. Fold change plot of significantly different metabolites. (**A**) positive ion model; (**B**) negative ion model; up-regulation and down-regulation are shown by red and green. The different colored words represent different superclasses of metabolites.

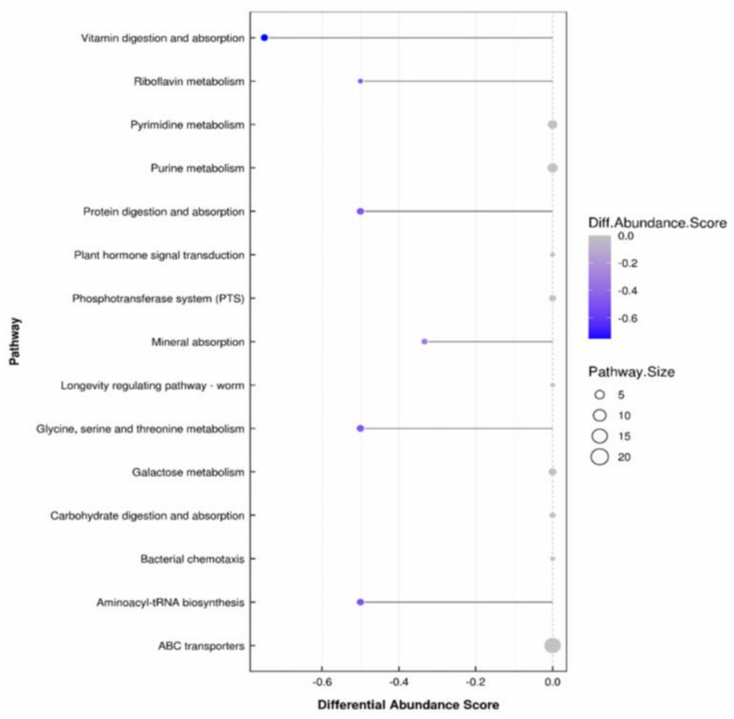

Figure S3. Differential abundance score. The x-axis is DA score, which represents total changes for all metabolites in a metabolic pathway; plus means up-regulation and minus means down-regulation. The length of the line represents the absolute Da score, and the dot size of the end point of the line represents the number of metabolites in that pathway. Shades of color of point are proportional to DA score.

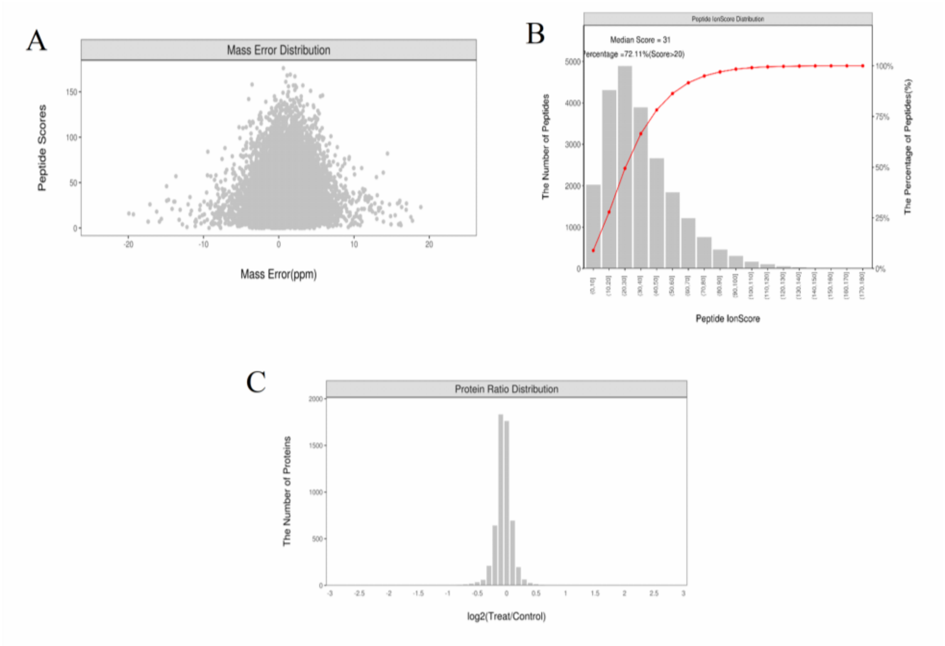

Figure S4. QC of proteome. (**A**) Mass error distribution. (**B**) Ion score distribution. (**C**) Ratio distribution between treat and control.

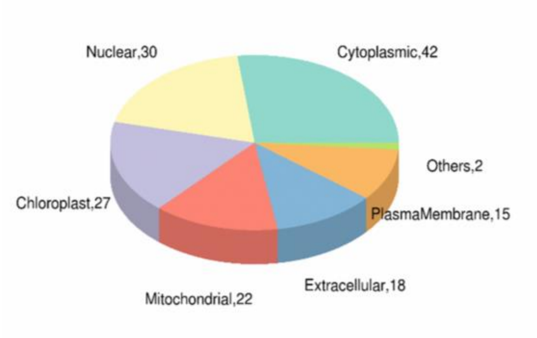

Figure S5. Subcellular localization statistic of DEPs. Number represents number of DEPs.

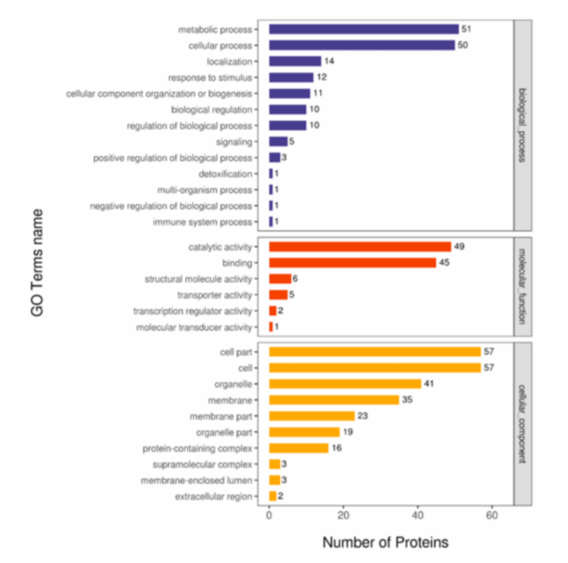

Figure S6. GO statistic of DEPs. Biological process, molecular function and cellular component were distinguished by different colors.

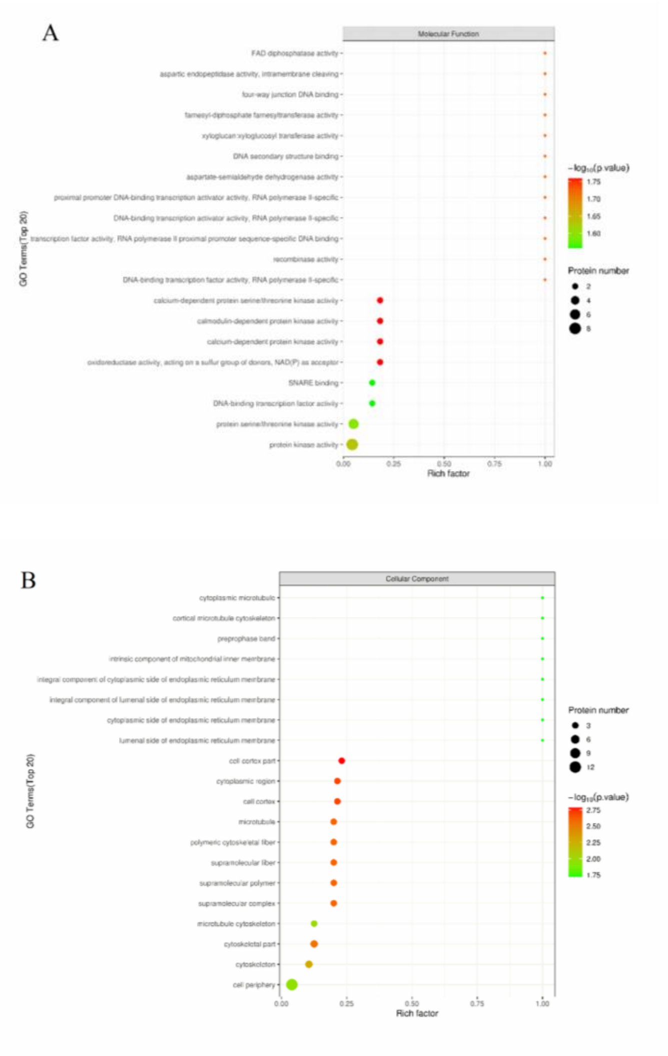

Figure S7. GO enrichment analysis of proteome. (**A**) Molecular function; (**B**) cell component. Abscissa is rich factor defined as ratio of DEP number, and the number of genes was annotated in this pathway; point size represents DEP number; p value shown by color from green to red.

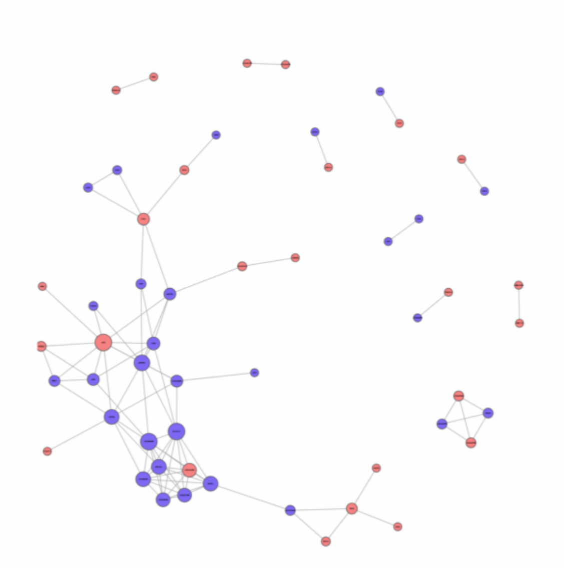

Figure S8. Protein–protein interaction network. Circle nodes indicate differentially expressed proteins, and lines indicate protein–protein interactions. Red indicates up-regulated protein, and blue is down-regulated protein. Circle size indicates the degree of protein connectivity.
